# Tuberculosis Drug Susceptibility, Treatment, and Outcomes for Belarusian HIV-Positive Patients with Tuberculosis: Results from a National and International Laboratory

**DOI:** 10.1155/2021/6646239

**Published:** 2021-04-02

**Authors:** Daria N. Podlekareva, Dorte Bek Folkvardsen, Alena Skrahina, Anna Vassilenko, Aliaksandr Skrahin, Henadz Hurevich, Dzmitry Klimuk, Igor Karpov, Jens D. Lundgren, Ole Kirk, Troels Lillebaek

**Affiliations:** ^1^CHIP, Rigshospitalet, University of Copenhagen, Denmark; ^2^International Reference Laboratory of Mycobacteriology, Statens Serum Institut, Copenhagen, Denmark; ^3^Republican Scientific and Practical Center for Pulmonology and TB, Minsk, Belarus; ^4^Belarusian State Medical University, Minsk, Belarus; ^5^Department of Infectious Diseases, Rigshospitalet, University of Copenhagen, Denmark; ^6^Global Health Section, Department of Public Health, University of Copenhagen, Denmark

## Abstract

**Background:**

To cure drug-resistant (DR) tuberculosis (TB), the antituberculous treatment should be guided by *Mycobacterium tuberculosis* drug-susceptibility testing (DST). In this study, we compared conventional DST performed in Minsk, Belarus, a TB DR high-burden country, with extensive geno- and phenotypic analyses performed at the WHO TB Supranational Reference Laboratory in Copenhagen, Denmark, for TB/HIV coinfected patients. Subsequently, DST results were related to treatment regimen and outcome.

**Methods:**

Thirty TB/HIV coinfected patients from Minsk were included and descriptive statistics applied.

**Results:**

Based on results from Minsk, 10 (33%) TB/HIV patients had drug-sensitive TB. Two (7%) had isoniazid monoresistant TB, 8 (27%) had multidrug-resistant (MDR) TB, 5 (17%) preextensive drug-resistant (preXDR) TB, and 5 (17%) had extensive drug-resistant (XDR) TB. For the first-line drugs rifampicin and isoniazid, there was DST agreement between Minsk and Copenhagen for 90% patients. For the second-line anti-TB drugs, discrepancies were more pronounced. For 14 (47%) patients, there were disagreements for at least one drug, and 4 (13%) patients were classified as having MDR-TB in Minsk but were classified as having preXDR-TB based on DST results in Copenhagen. Initially, all patients received standard anti-TB treatment with rifampicin, isoniazid, pyrazinamide, and ethambutol. However, this was only suitable for 40% of the patients based on DST. On average, DR-TB patients were changed to 4 (IQR 3-5) active drugs after 1.5 months (IQR 1-2). After treatment adjustment, the treatment duration was 8 months (IQR 2-11). Four (22%) patients with DR-TB received treatment for >18 months. In total, sixteen (53%) patients died during 24 months of follow-up.

**Conclusions:**

We found high concordance for rifampicin and isoniazid DST between the Minsk and Copenhagen laboratories, whereas discrepancies for second-line drugs were more pronounced. For patients with DR-TB, treatment was often insufficient and relevant adjustments delayed. This example from Minsk, Belarus, underlines two crucial points in the management of DR-TB: the urgent need for implementation of rapid molecular DSTs and availability of second-line drugs in all DR-TB high-burden settings. Carefully designed individualized treatment regimens in accordance with DST patterns will likely improve patients' outcome and reduce transmission with drug-resistant *Mycobacterium tuberculosis* strains.

## 1. Introduction

Tuberculosis (TB) continues to be a major public health threat, especially due to drug-resistant TB (DR-TB) [1]. Of special concern are the increasing rates of HIV-associated TB and multidrug-resistant TB (MDR-TB) in eastern Europe. In this region, the rates of MDR-TB are among the highest in the world, ranging from 12-40% among new TB cases to 30-70% among previously treated [1, 2]. Belarus holds some of the highest MDR-TB rates in the region (app. 40% and 70% among new and previously treated patients, respectively) [1, 3]. While the first is an indicator of poor infectious control and ongoing *Mycobacterium tuberculosis* (*Mtb*) transmission, the latter indicates inappropriate management where new or additional resistance develops during treatment.

In eastern Europe, the HIV-positive population is particularly vulnerable to TB acquisition due to overlapping risk groups and a generally higher risk of TB disease due to more pronounced immunodeficiency. As a result, the prevalence of coinfection with *Mtb* and HIV is increasing in this region [1, 4, 5].

Diagnosis of TB and determination of drug-susceptibility patterns using conventional culture-based phenotypic methods (i.e., culture on either solid or liquid media) can be time consuming (up to few months), which may compromise timely initiation of active treatment. This is especially problematic in settings with a high prevalence of MDR-TB. Access to rapid DNA-based genotypic drug-susceptibility tests (DST) may allow for rapid adjustment of therapy according to resistance patterns, potentially helping to improve clinical outcomes and reducing complications for the individual patients, as well as helping to reduce transmission with resistant *Mtb* strains in society.

Treatment outcome for TB/HIV patients in eastern Europe, especially those with MDR-TB, is poor, with a reported one-year mortality rate of approximately 30% and higher [6, 7]. Management of MDR-TB, particularly in the context of HIV infection, can be complicated. It is crucial to design individualized drug-regimens based on specific *Mtb* susceptibility patterns as fast as possible. In situations, where susceptibility patterns are not available and patients are treated empirically, there is a high risk of suboptimal treatment regimens with a limited number of active drugs, which may lead to drug resistance [8].

In this study, we explored the management of TB/HIV patients by comparing DST results from the Republican Scientific and Practical Center for Pulmonology and TB, Minsk, Belarus (Minsk), and the WHO TB Supranational Reference Laboratory (Copenhagen) in Copenhagen, Denmark. We aimed then to assess adequacy of used treatment regimens and patients' survival according to the DST patterns. In addition, we compare phenotypic DST results with rapid DNA-based genotypic DST and whole-genome sequencing (WGS) results and describe the genomic epidemiology of the involved *Mtb* strains.

## 2. Methods

As part of the international prospective TB : HIV cohort study (https://www.chip.dk), consecutive HIV-positive patients from Minsk with TB diagnosis between 01/01/2011 and 31/12/2013 were identified [9]. Demographic and clinical data were collected on standardized case report forms at the date of TB diagnosis (baseline), and at 6, 12, and 24 months thereafter. Specific information on TB treatment was collected, as were the results of locally performed DSTs for *Mtb*. Participants were followed until two years after TB diagnosis, date of death, or loss to follow-up. Ethical approval was obtained in accordance with local rules and legislations.

### 2.1. Laboratory Methods

For first-line anti-TB drugs, the DST assays used in Minsk were conventional phenotypic methods on solid Löwenstein-Jensen (LJ) media and liquid Mycobacteria Growth Indicator Tube (MGIT) between 2011 and 2013. For the second-line drugs, DST was performed both phenotypically on MGIT and for some patients genotypically by GenoType MTBDR*sl* (Hain Lifescience, Nehren, Germany) (limited availability in 2011-2015).

The *Mtb* cultures on a solid LJ media were stored locally and subsequently shipped to the SRL according to the international regulations for shipment of class III material (Category A material according to the IATA Dangerous Goods Regulations, https://www.iata.org/en/programs/cargo/dgr/), for phenotypic and genotypic DST. In Copenhagen, all strains were subcultured in Dubos before performing DST. Initially, phenotypic DST performed for the four first-line drugs: isoniazid (H), rifampicin (R), ethambutol (E), and pyrazinamide (Z). Only in case of any first-line resistance was DST for second-line drugs performed. These included aminoglycosides (streptomycin (S), amikacin (Am), kanamycin (Km), and capreomycin (Cm)), fluoroquinolones (moxifloxacin (Mfx), ofloxacin (Ofx)), ethionamide (Eto), and linezolid (Lzd). Phenotypic DST was performed on MGIT 960, and all drugs were provided by the manufacturer. The MGIT 960 SIRE kit (Becton Dickinson) contained lyophilized vials with low (critical) and high concentrations for first-line drugs. For 2nd-line drugs, recommended critical concentrations were used [10]. The results were reported as sensitive or resistant by the system. When a “resistant” result was obtained, the vial was checked for purity and verified by retesting.

The genotypic DST was performed by using the line probe assay (LPA) GenoType MTBDR*plus* and *sl* (Hain Lifescience, Nehren, Germany), the latter if any phenotypic resistance to first-line drugs was detected, or if subculture failed to grow. Analysis and interpretation of results were carried out according to manufacturer's instructions (https://www.hain-lifescience.de/en/). Among first-line drugs, resistance to isoniazid and rifampicin was determined by detection of mutations in inhA/katG genes and in rpoB gene of *Mtb* strains, respectively. Among second-line drugs, resistance to fluoroquinolones was determined by mutations in gyrA and gyrB genes; and resistance to aminoglycosides by was determined by detection of mutations in rrs and eis genes [11].

Whole-genome sequencing (WGS) was performed for all *Mtb* culture samples as previously described [12]. The resulting FastQ files were uploaded to PhyResSe, a Phylo-Resistance Search Engine used to search for mutations conferring resistance (https://bioinf.fz-borstel.de/mchips/phyresse/) [13].

In addition to the mutations listed above, WGS allowed for additional genotypic susceptibility testing for the following drugs: ethambutol (by detection of mutations in the embB gene), pyrazinamide (mutations in the pncA and rpsA genes), streptomycin (mutations in rpsL gene), ethionamide (mutations in the mshA and ethA genes), linezolid (mutations in the rplC gene), and para-aminosalicylic acid (PAS) (mutation in thyA). All strains with a coverage > 25x were included. Sequences have been deposited in the European Nucleotide Archive under project accession number PRJEB38234.

### 2.2. Statistics and Study Definitions

**Figure 1:**
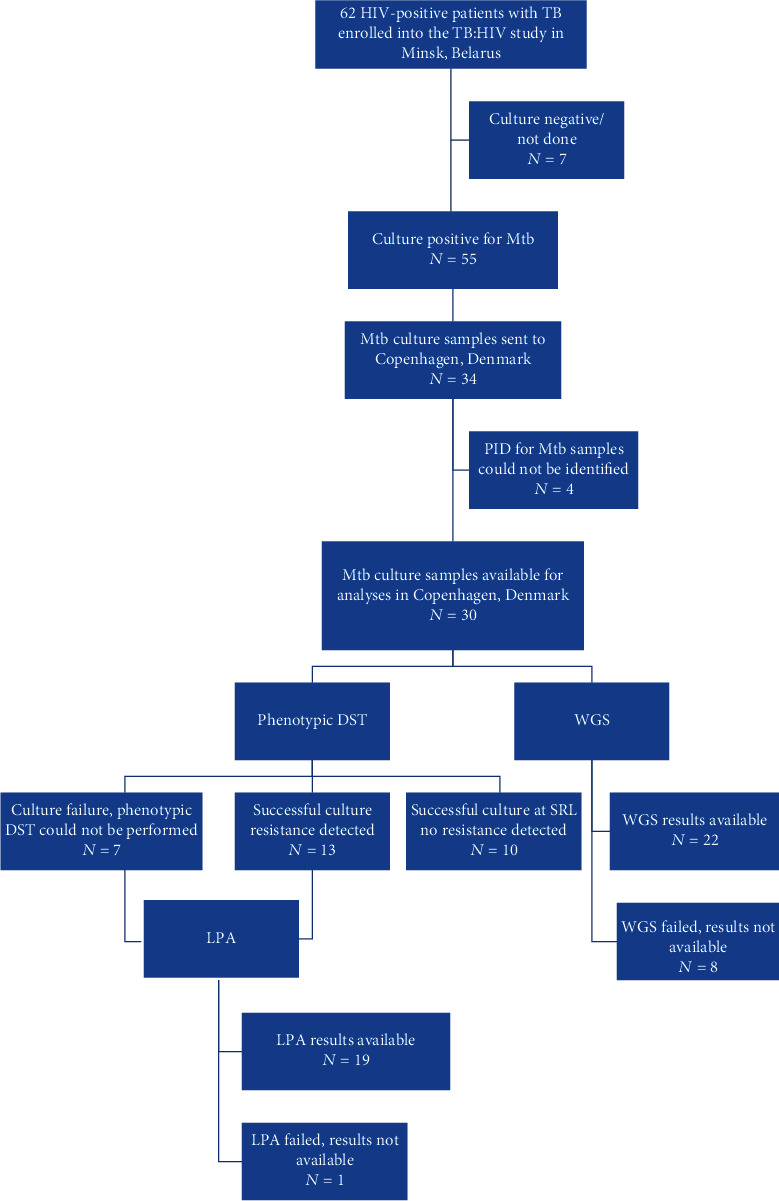
Flow chart of TB/HIV patients from Minsk, Belarus, enrolled into the TB : HIV study during 2011-2013, who have *Mycobacterium tuberculosis* (*Mtb*) culture samples available and with various drug susceptibly tests performed on these samples. Abbreviations: DST = drug-susceptibility testing; LPA = line probe assay; WGS = whole-genome sequencing. Patients were enrolled and followed up from the Republican Scientific and Practical Center for Pulmonology and TB, Minsk, Belarus. *Mtb* cultures were sent to the WHO TB Supranational Reference Laboratory, Copenhagen, Denmark, where drug-susceptibility testing was performed using phenotypic, LPA, and WGS methods.

TB/HIV patients from Minsk (*n* = 62) were stratified according to the availability of *Mtb* culture samples, and descriptive statistics were used to compare baseline demographic and clinical characteristics. Further, the group with *Mtb* samples available (*n* = 30) was characterized in detail according to resistance and treatment patterns (Figure 1). DST results reported from Minsk were compared with phenotypic DST, LPA, and WGS performed in Copenhagen.

Patients were grouped according to the following resistance patterns: drug-sensitive (DS) TB, MDR-TB, preXDR-TB, and XDR-TB (Table 1). In Copenhagen, the *Mtb* in samples were considered resistant if resistance was detected in any of the analyses (either phenotypic, LPA, or WGS). Specific treatment patterns were analysed for each patient and included initial anti-TB treatment and any consecutive changes.

## 3. Results

A total of 62 patients were enrolled from Minsk. Of those, 55 (89%) were *Mtb* culture positive. Samples from 30 patients (55% of all culture positive) were stored locally and sent to Copenhagen (Figure 1).  Table 2 presents baseline clinical and demographic characteristics of patients with/without *Mtb* culture samples and shows that these two groups are comparable. The majority of patients in both groups were young males with a history of injected-drug use (IDU), imprisonment, and/or excessive alcohol consumption. Even though the majority of patients were diagnosed with HIV infection several years before TB diagnosis, only a small proportion were on antiretroviral therapy (ART) at time of TB diagnosis, and the majority had low CD4 cell counts (Table 2).

In Copenhagen, 7 of 30 samples (23%) failed to grow. For samples fully susceptible based on phenotypic DST, genotypic DST was not performed, except for WGS, which was performed for all samples but failed for 8 (27%). For samples where phenotypic resistance was detected, or there was no culture growth, an LPA analysis was performed (Flowchart).

### 3.1. Drug-Susceptibility Test Patterns

DST results, provided by Minsk and Copenhagen, are presented in Tables 3 and 4. DST results for all four first-line drugs from Minsk were available, except for 11 cases, where DST for pyrazinamide was not reported. DST for second-line drugs in Minsk included aminoglycosides (S, Amk/Km, and Cm), fluoroquinolones (Ofx, Lfx), Eto/Pto, Cs, and PAS. DSTs for Mfx and Lzd were not performed in Minsk during the study period, whereas DST for Lfx, Cs, and PAS were not performed in Copenhagen (Table 3).

Good agreement between the two laboratories was observed comparing the results of the phenotypic DST for first-line drugs (Tables 3 and 4): 18 out of 23 cases with available results were identical (78%). Two patients (PIDs 13 and 14) were found to have MDR-TB in Minsk but DS-TB in Copenhagen, and one patient (PID 26) had discrepancies in rifampicin phenotypic testing: resistant in Minsk and sensitive in Copenhagen. Another patient (PID 18) was found to be infected with *Mtb* resistant to ethambutol and pyrazinamide in Minsk and sensitive in Copenhagen, and PID 15 had *Mtb* resistant to ethambutol in Minsk and sensitive in Copenhagen (Table 3).

Among phenotypic DSTs for second-line drugs, discrepancies were noticed for 7 out of 11 *Mtb* samples, where *Mtb* was sensitive for these drugs in Minsk but resistant in Copenhagen. Few other minor discrepancies were observed in DSTs for aminoglycosides (Table 3).

According to the susceptibility patterns obtained from Minsk, *Mtb* strains from 10 (33.3%) patients were classified as fully DS-TB; 2 (6.7%) as isoniazid monoresistant TB; 8 (26.7%) as MDR-TB; 5 (16.7%) as preXDR-TB, and 5 (16.7%) as XDR-TB (Table 4). In Copenhagen, DS-TB was diagnosed in 12 patients (40.0%, *p* = 0.79).

Comparing results from Minsk and Copenhagen laboratories for patients with drug-resistant TB, MDR-TB was found in 8 of 30 (26.7%) vs. 3 of 30 (10.0%), *p* = 0.18; preXDR-TB was found in 5 of 30 (16.7%) vs. 8 of 30 (30.0%), *p* = 0.36; and XDR-TB was found in 5 of 30 (16.7%) vs. 7 of 30 (13.3%), *p* = 1.0, respectively (Table 4).

### 3.2. TB Treatment Regimens and Survival

**Figure 2:**
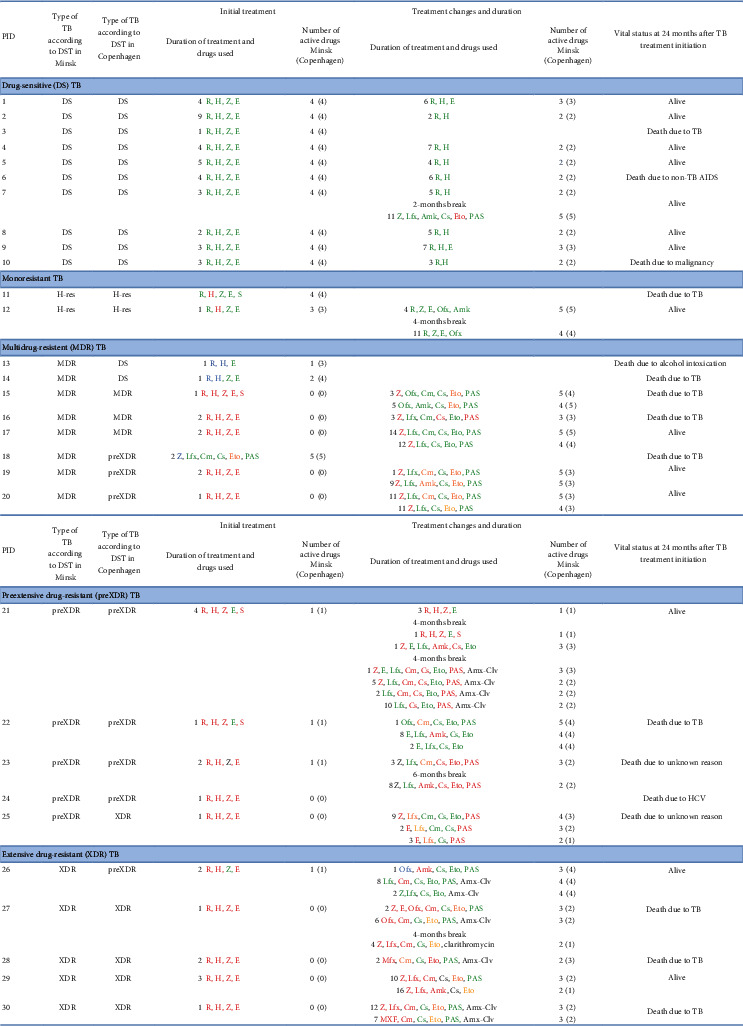
Individual TB treatment patterns and vital status at 24 months after TB diagnosis for 30 TB/HIV patients from Minsk, Belarus according to the results of *Mycobacterium tuberculosis* (*Mtb*) drug-susceptibility testing performed at two different laboratories (Belarus vs. Denmark). Abbreviations: Am = amikacin; Amx-Clv = amoxicillin-clavulanic acid; Cm = capreomycin; Cs = cycloserine; E = ethambutol; Eto = ethionamide; H = isoniazid; Km = kanamycin; Lfx = levofloxacin; Lzd = linezolid; Mfx = moxifloxacin; Ofx = ofloxacin; PAS = *p*-aminosalicylic acid; Pto = prothionamide; R = rifampicin; S = streptomycin; Z = pyrazinamide; PID = patient's identification number. In Minsk, DST was performed at the Republican Scientific and Practical Center for Pulmonology and TB, Minsk, Belarus. In Copenhagen, DST was performed at the WHO TB Supranational Reference Laboratory, Copenhagen, Denmark. Number in front of drug combination means number of months the drug combination was used. A drug was included in the analysis, if it was used for >15 days. Green = *Mtb* was sensitive to a drug. Red = *Mtb* was resistant to a drug. Orange = *Mtb* was sensitive to a drug by DST analysis in Minsk and resistant in Copenhagen. Blue = *Mtb* was resistant by DST analysis in Minsk and sensitive in Copenhagen. In case of discrepancies between phenotypic and genotypic tests within laboratory in Copenhagen, the worst result (i.e. resistance) was considered. DS = Drug sensitive TB; *Mtb* sensitive to all first-line anti-TB drugs. Mono-resistant TB = *Mtb* resistant to one first-line anti-TB drug only. MDR-TB = multidrug resistant TB; *Mtb* resistant to at least both isoniazid and rifampicin. preXDR-TB = preextensive drug resistance; *Mtb* resistant to isoniazid and rifampicin and either to any fluoroquinolone or a second-line injectable agent but not to both. XDR-TB = extensive drug resistance; *Mtb* resistant to isoniazid and rifampicin and to any fluoroquinolone and to at least one of three second-line injectable drugs.

Patterns of anti-TB treatment for each patient are presented in Figure 2. Twenty-eight (93%) patients initiated treatment with a standard four-drug first-line regimen (RHZE), and of these, four (14%) had streptomycin added. All patients with DS-TB according to Minsk results (*n* = 10) were treated with standard first-line anti-TB drugs, but for various durations, including prolonged intensive phases of treatment (Figure 2). Median treatment duration for DS-TB was 9.5 months (IQR 7.3-10.0 months). Three patients with DS-TB died: one within the first month of treatment where TB was indicated as the cause of death, and two due to non-TB related causes after completion of anti-TB treatment.

Among the two patients with isoniazid monoresistant TB, 1 died due to TB 3 days after initiation of the RHZES regimen.

Patients with at least MDR-TB (*n* = 18) according to Minsk data switched from the initial first-line to a second-line treatment after a median of 1 month (IQR 1-2 months). Second-line regimens were standardized and included one fluoroquinolone (Ofx or Lfx), one aminoglycoside (mainly Cm, and to a rarer extent, Am), Cs, Eto, and PAS. Pyrazinamide was also commonly used in second-line regimens despite resistance being reported. Second-line regimens contained a median of 6 drugs. However, the median number of active drugs was 4 (IQR 3-5) according to the national DST results, and 3 (IQR 2-4) according to the DST results obtained in Copenhagen. After the first treatment adjustment, 8 patients according to Minsk and 10 patients according to Copenhagen DST results received 3 or less active drugs. Further treatment switches were directed on either exchange drugs within the same drug class (e.g., Ofx to Lfx) or on a reduction in the number of drugs (also a reduction in the number of active drugs) (Figure 2). Median treatment duration with second-line drugs for patients with at least MDR-TB was 12 months (IQR 10-21). Twelve patients (67%) with at least MDR-TB died within 24 months of treatment initiation: 8 (67%) due to TB and 4 (33%) due to some other reasons (Figure 2). For those, who were alive at 24 months (*n* = 6), treatment duration ranged from 10 to 26 months.

### 3.3. Lineages

Half of the successfully genotyped strains belonged to lineage 2 (Beijing) (*n* = 11/22), and of those, 7 (63%) had at least MDR-TB and 7 died. Among people with other lineages, 4 (36%) had at least MDR-TB and 5 (45%) died. The small numbers did not allow for further analyses.

## 4. Discussion

Comparing *Mtb* DST between the national laboratory in Minsk, a middle-income MDR-TB high-prevalence setting, and the WHO TB Supranational Reference Laboratory in Copenhagen, we found a good level of concordance for 1st line anti-TB drugs, whereas there was some discordance for second-line drugs. In addition, several issues in the management of TB/HIV patients in Minsk were identified, particularly in the management of patients with DR-TB. In Copenhagen, quality assurance of *Mtb* DST from Minsk included both phenotypic and genotypic tests, namely LPA and WGS. In general, the DST performed in Minsk were reliable and results of phenotypic DSTs for first-line drugs for patients with DS-TB were nearly identical between the two laboratories except for two patients who were reported as having MDR-TB locally, but DS-TB in Copenhagen. For pyrazinamide and ethambutol, phenotypic DST is usually challenging, but DST results for these two drugs were very consistent and similar between laboratories with only few discrepancies [14].

All patients with DS-TB were treated with four first-line drugs during intensive phase, followed by 2-3 drugs in the continuation phase, generally in concordance with local guidelines recommending longer treatment duration in case of HIV coinfection at that time.

At the time of data collection, phenotypic DST for the main second-line drugs (fluoroquinolones and aminoglycosides) was available and was performed for all 30 patients in Minsk. Although phenotypic DST for second-line drugs was not performed in Copenhagen for patients with DS-TB, DST results from Minsk were very consistent with WGS results in Copenhagen. The discrepancies in resistance patterns for second-line drugs were therefore observed among patients with DR-TB, but these also included discrepancies between phenotypic, LPA, and WGS internally within the Copenhagen laboratory. This discrepancy between different methods in Copenhagen was primarily, but not exclusively, observed for aminoglycosides. While LPA, and to some extent WGS, predominately include well-documented mutations, there might be less known mutations that are expressed phenotypically, but not yet captured by the genetic analyses. In addition, novel mutations continue to arise or are discovered/described.

In our study, the majority of patients with DR-TB spent at least one month on empirical RHZE-containing treatment prior to switching to a second-line regimen. Despite DST availability for both first- and second-line drugs, all patients with DR-TB received standard second-line treatment, including those with (pre)XDR. Thus, treatment was suboptimal for many patients with (pre)XDR-TB. Using Copenhagen results, patients with DR-TB received a smaller number of active drugs. Although the number of drugs was sufficient for some patients (i.e., 4 drugs), the regimens seemed to be weak in their effectiveness, and availability of early DST results could potentially have allowed avoidance of ineffective and more toxic drugs [15]. Another important observation is that despite of DST patterns becoming available during treatment, patients were not switched to a more effective regimen. This could reflect reduced drug availability at the time of study and may have subsequently improved [16, 17]. Bedaquiline, for example, was not available at the time of the study, but it is now.

At the time of data collection, the recommended treatment duration for MDR-TB was 18-24 months [18]. The median duration of treatment of MDR-TB in our study was difficult to assess, as the majority of patients died while on treatment. More recently, shorter treatment durations have become a possibility for some patients with MDR-TB only, which underlines the importance of rapid DSTs for second-line drugs to rule out (pre)XDR-TB [19, 20].

DST is essential in the management of TB patients in settings with a high prevalence of DR-TB. Ideally, DST should be performed prior to initiation of TB therapy, especially in patients with a previous history of TB or known exposure for DR-TB, to guide clinical management. The use of rapid molecular assays reduces the time for drug resistance diagnostic to just a few days, and may help not only to guide treatment but also to control the ongoing TB transmission. This is of particular importance in settings with a high prevalence of MDR-TB, where rapid DST for second-line drugs can help to exclude resistance and allow for treatment adjustments.

In a few cases, we observed a discrepancy between the phenotypic and genotypic resistance patterns. This is a phenomenon, which has been described previously and underlines the need for both methods at present [14, 21]. This is because not all molecular mechanisms of drug resistance are known, and new genes conferring resistance are continuously being described [12, 22, 23]. On the other hand, several studies have documented that some clinically relevant resistance mutations could be overlooked in phenotypic DSTs [21, 24]. Thus, while rapid molecular DSTs for *Mtb* is now paramount in TB diagnostics, especially in settings with high prevalence of DR-TB, phenotypic DST still plays an important role in the management of TB, especially for second-line and new drugs.

Patients in our study initiated their TB treatment between 2011 and 2013. At that time, WHO guidelines already recommended rapid molecular DSTs, such as GeneXpert and LPA, to determine susceptibility for rifampicin and isoniazid prior to treatment initiation [18]. However, this was not yet implemented in Minsk. Currently, WHO recommends GeneXpert as the primary diagnostic tool, and if rifampicin resistance is detected, LPA for second-line drugs should be performed.

In recent years, new molecular methods applicable directly on primary specimens have become available. For example, we now have FlouroType MTBDR (Hain Lifescience, Nehren, Germany) and Deeplex Myc-TB (GenoScreen, Lille, France). These may help to further reduce the time until targeted personalized treatment can be initiated [25].

Half of the *Mtb* strains successfully genotyped in our study were of the Beijing spoligotype, a lineage 2 strain (11/22), which is in accordance with previous results from Minsk [26]. It has been speculated that some lineages, including linage 2, may be more virulent than others [27]. As none of the study strains were identical, we saw no indications of recent *Mtb* transmission between patients (data not shown).

Regarding study limitations, the sample size was small resulting in limited statistical power. However, our data on the prevalence of MDR-TB is representative for Belarus (and Minsk), where the prevalence of MDR-TB is approximately 40% [1]. In addition, all patients in the study were TB/HIV coinfected; thus, it was not possible to assess the influence of HIV on *Mtb* transmission and development of resistance. It is also worth to noting that MDR-TB treatment guidelines in Belarus have recently been revised, and they are now recommending oral regimens and avoidance of the most toxic drugs, particularly aminoglycosides. Further, new drugs (e.g., linezolid, clofazimine, bedaquiline, and delamanid) have now become available in Belarus.

To the best of our knowledge, this is the first study providing detailed description of anti-TB treatment for individual TB patients according to *Mtb* drug resistance patterns. It is a good example for TB clinicians elsewhere on the importance of rapid DSTs and concordance between resistance patterns and timely prescribed adequate anti-TB treatment. Overall, outcomes of patients with DR-TB are known to be poor, with a success rate of approximately 50-60% [1, 5]. Studies have shown that treatment success improves essentially when adequate treatment regimens (in terms of efficacy and number of effective drugs) are used [28].

In conclusion, we found some discordances between phenotypic DST results in Belarus and an international reference laboratory in Copenhagen. This was especially true for second-line drugs, compromising early identification and initiation of individualized treatment of patients with DR-TB. Our results advocate for wide implementation and validation of rapid DSTs in all DR-TB high-burden areas with simultaneous wide availability of drugs for the treatment of drug-resistant TB. Future studies are encouraged to follow-up on resistance and treatment patterns, drugs tolerability, and patient outcomes.

## Figures and Tables

**Table 1:** Definitions for anti-TB drug resistance [29, 30].

Resistance terminology	Definition
Drug-sensitive TB (DS-TB)	*Mtb* sensitive to all first-line anti-TB drugs
Monoresistant TB	*Mtb* resistant to one first-line anti-TB drug only
Drug-resistant TB (DR-TB)	At least MDR-TB, combined terminology for any of the below definitions
Multidrug-resistant TB (MDR-TB)	*Mtb* resistant to at least both isoniazid and rifampicin
Preextensive drug-resistant TB (preXDR-TB)	*Mtb* resistant to isoniazid and rifampicin and either any fluoroquinolone *or* a second-line injectable agent but not both
Extensive drug-resistant TB (XDR-TB)	*Mtb* resistant to isoniazid and rifampicin and to any fluoroquinolone *and* to at least one of three second-line injectable drugs (capreomycin, kanamycin, and amikacin)

Mtb = *Mycobacterium tuberculosis.*

**Table 2:** Baseline characteristics of 62 TB/HIV coinfected patients from Minsk, Belarus, stratified according to availability of *Mycobacterium tuberculosis* (Mtb) culture sample.

Total		Sample yes, *n* (%)	Sample no, *n* (%)	*p*
		**30**	**32**	
Gender	Male, *n* (%)	22 (73)	28 (88)	0.206
Age	Years, median (IQR)	37 (30-41)	35 (32-42)	0.789
TB/HIV risk factors	Ever injecting drug use, *n* (%)	19 (63)	24 (75)	0.319
	History of imprisonment, *n* (%)	5 (17)	12 (38)	0.090
	History of excess alcohol consumption, *n* (%)	19 (63)	15 (47)	0.213
*Mtb* culture positive	Yes, *n* (%)	30 (100)	25 (78)	0.0015
MDR-TB	Yes, *n* (%)	18 (60)	16 (50)	0.456
TB disease	Disseminated, *n* (%)	12 (40)	6 (19)	0.060
Hepatitis C∗	Ever, *n* (%)	24 (80)	23 (72)	0.454
HIV duration prior to TB	Months, median (IQR)	88 (44-136)	67 (25-120)	0.535
Antiretroviral therapy at baseline	Yes, *n* (%)	15 (50)	14 (44)	0.622
CD4 count	Cells/mm^3^, median (IQR)	85 (22-171)	126 (57-310)	0.097

Abbreviations: baseline = date of TB diagnosis; IQR = interquartile range; *n* = number; MDR-TB = multidrug-resistant TB; Mtb = *Mycobacterium tuberculosis*; TB = tuberculosis; ^∗^Hepatitis C antibody positive.

**Table 3:** Individual results of *Mycobacterium tuberculosis* (Mtb) drug-susceptibility testing performed in two different laboratories (Belarus vs. Denmark) for 30 TB/HIV patients from Minsk, Belarus.

PID	Place of analysis		Isoniazid	Rifampicin	Ethambutol	Pyrazinamide	Streptomycin	Amikacin	Kanamycin	Capreomycin	Moxifloxacin	Ofloxacin	Levofloxacin	E-/prothionamide	Linezolid	Cycloserine	*p-*Amino salicylic acid	Lineage
Drug-sensitive TB																		
1	Minsk		S	S	S		S	S	S	S		S				S	S	
	Copenhagen	Pheno	S	S	S	S												
		LPA																
		WGS	S	S	S	S	S	S	S	S	S	S						Beijing
2	Minsk		S	S	S		R	S	S	S		R		S		S	S	
	Copenhagen	Pheno	S	S	S	S												
		LPA																
		WGS	S	S	S	S	S	S	S	S	S	S						EAS
3	Minsk		S	S	S		S			S		S		S		S	S	
	Copenhagen	Pheno	S	S	S	S												
		LPA																
		WGS	S	S	S	S	S	S	S	S	S	S						Beijing
4	Minsk		S	S	S		S	S	S	S		S		S		S	S	
	Copenhagen	Pheno	S	S	S	S												
		LPA																
		WGS	S	S	S	S	S	S	S	S	S	S						Beijing
5	Minsk		S	S	S	S	S	S	S	S		S		S		S	S	
	Copenhagen	Pheno	S	S	S	S												
		LPA																
		WGS	S	S	S	S	S	S	S	S	S	S						EAS
6	Minsk		S	S	S	S												
	Copenhagen	Pheno	S	S	S	S												
		LPA																
		WGS	S	S	S	S	S	S	S	S	S	S						EAS
7	Minsk		S	S	S	S	S	S	S	S		S		S		S	S	
	Copenhagen	Pheno	S	S	S	S												
		LPA																
		WGS	S	S	S	S	S	S	S	S	S	S						Ural
8	Minsk		S	S	S	S	R	S	S	S		S	S	S		S	S	
	Copenhagen	Pheno	Failed															
		LPA	S	S				S	S	S		S						
		WGS	Failed															
9	Minsk		S	S	S	S		S	S	S			S					
	Copenhagen	Pheno	Failed															
		LPA	S	S				S	S	S	S	S						
		WGS	Failed															
10	Minsk		S	S	S	S	S	S	S	S			S	S		S	S	
	Copenhagen	Pheno	S	S	S	S												
		LPA																
		WGS	S	S	S	S	S	S	S	S	S	S						EAS
Monoresistant TB																		
11	Minsk		R	S	S	S	S	S	S	S		S		S		S	S	
	Copenhagen	Pheno	R	S	S	S	S	S	S	S	S	S		S	S			
		LPA	R	S				S	S	S	S	S						
		WGS	R	S	S	S	S	S	S	S	S	S						Beijing
12	Minsk		R	S	S		S	S	S			S						
	Copenhagen	Pheno	R	S	S	S	S	S	S	S	S	S		S	S			
		LPA	R	S														
		WGS	R	S	S	S	S	S	S	S	S	S						LAM
Multidrug-resistant TB																		
13	Minsk		R	R	S		S	S	S									
	Copenhagen	Pheno	S	S	S	S												
		LPA																
		WGS	S	S	S	S	S	S	S	S	S	S						EAS
14	Minsk		R	R	S	S	R	S	S	S			S					
	Copenhagen	Pheno	S	S	S	S												
		LPA	N/A															
		WGS	Failed															
15	Minsk		R	R	R	R	R	S	S	S		S		S		S	S	
	Copenhagen	Pheno	R	R	S	R	R	S	S	S	S	S		R	S			
		LPA	R	R				S	S	S	S	S						
		WGS	R	R	R	R	R	S	S	S	S	S						Beijing
16	Minsk		R	R	R		R	S	S	S			S	S		R	R	
	Copenhagen	Pheno	R	R	R	R	R	NG										
		LPA	R	R				S	S	S	S	S						
		WGS	R	R	R	S	R	S	S	S	S	S						LAM
17	Minsk		R	R	R	R	R	S	S	S		S	S	S		S	S	
	Copenhagen	Pheno	Failed															
		LPA	R	R				S	S	S	S	S						
		WGS	Failed															
18	Minsk		R	R	R	R	R	S	S	S		S		S		S	S	
	Copenhagen	Pheno	R	R	S	S	R	S	R	S	S	S		R	S			
		LPA	R	R				S	S	S	S	S						
		WGS	R	R	R	S	R	S	S	S	S	S						Beijing
19	Minsk		R	R	R		R	S	S	S			S	S		S	S	
	Copenhagen	Pheno	R	R	R	R	R	S	S	S	S	S		R	S			
		LPA	R	R				R	R	R	S	S						
		WGS	R	R	R	R	R	S	S	S	S	S						LAM
20	Minsk		R	R	R	R	R	S	S	S		S		S		S	S	
	Copenhagen	Pheno	R	R	R	R	R	S	R	S	S	S		R	S			
		LPA	R	R				R	R	R	S	S						
		WGS	R	R	R	R	R	S	S	S	S	S						LAM
Preextensive drug-resistant TB																		
21	Minsk		R	R	S	R	R	R	R	R			S	S		R	R	
	Copenhagen	Pheno	R	R	S	R	R	R	R	R	S	S		S	S			
		LPA	R	R				R	R	R	S	S						
		WGS	Failed															
22	Minsk		R	R	S		R	R	R	S		S		S		S	S	
	Copenhagen	Pheno	Failed															
		LPA	R	R				R	R	R	S	S						
		WGS	Failed															
23	Minsk		R	R	R			R	R	S			S	R		R	R	
	Copenhagen	Pheno	Failed															
		LPA	R	NC				R	R	R	S	S						
		WGS	Failed															
24	Minsk		R	R	R		R	S	R	S		S		R		S	R	
	Copenhagen	Pheno	Failed															
		LPA	R	R				R	R	R	S	S						
		WGS	R	R	R	R	R	S	S	S	S	S						LAM
25	Minsk		R	R	R	R	R	S	S	S		R	S	S		S	R	
	Copenhagen	Pheno	R	R	R	R	R	S	R	S	R	R		S	S			
		LPA	R	R				S	S	S	R	R						
		WGS	R	R	R	R	R	S	S	S	R	R						Beijing
Extensive drug-resistant TB																		
26	Minsk		R	R	R	S	R	R	R	R		R		S		S	S	
	Copenhagen	Pheno	R	S	R	S	R	R	R	R	S	S		S	S			
		LPA	R	S				R	R	R	S	S						
		WGS	R	R	R	S	R	R	R	R	S	S						Beijing
27	Minsk		R	R	R	R	R	R	R	R		R		S		S	S	
	Copenhagen	Pheno	R	R	R	R	R	R	R	S	R	R		R	S			
		LPA	R	R				R	R	R	R	R						
		WGS	R	R	R	R	S	R	R	R	R	R						Beijing
28	Minsk		R	R	R	R	R	S	R	S		R	R	R		S	S	
	Copenhagen	Pheno	Failed															
		LPA	R	R				R	R	R	R	R						
		WGS	Failed															
29	Minsk		R	R	R	R	R	R	R			R		S			S	
	Copenhagen	Pheno	R	R	R	R	R	R	R	S	R	R		R	S			
		LPA	R	R				R	R	R	R	R						
		WGS	R	R	R	S	S	R	R	R	R	R						Beijing
30	Minsk		R	R	R	R	R	R	R	R			R	S		S	S	
	Copenhagen	Pheno	R	R	R	R	R	R	R	R	R	R		R	S			
		LPA	R	R				R	R	R	R	R						
		WGS	R	R	R	R	R	R	R	R	R	R						Beijing

Abbreviations: PID = patient's identification number. DST = drug susceptibility testing. LPA = line probe assay. Pheno = phenotypic resistance. R = resistant. S = sensitive. WGS = whole-genome sequencing. Genotypic resistance was determined by detection of mutations in the inhA/katG genes for isoniazid, in the rpoB gene for rifampicin, in the embB gene for ethambutol, and in the pncA gene for pyrazinamide. For second-line drugs, resistance was determined for a drug class by detection of the following mutations: aminoglycosides (amikacin, capreomycin, and kanamycin) in the rrs and eis genes; streptomycin in the rpsL gene; fluoroquinolones (moxifloxacin, levofloxacin, and ofloxacin) in the gyrA and gyrB genes. EAS = Euro-American superlineage, LAM = Latin American-Mediterranean lineage. In Minsk, DST was performed at the Republican Scientific and Practical Center for Pulmonology and TB, Minsk, Belarus. In Copenhagen, DST was performed at the WHO TB Supranational Reference Laboratory, Copenhagen, Denmark. Drug-sensitive TB = *Mtb* sensitive to all first-line anti-TB drugs. Monoresistant TB = *Mtb* resistant to one first-line anti-TB drug only. Multidrug resistant TB = *Mtb* resistant to at least both isoniazid and rifampicin. Preextensive drug resistance = *Mtb* resistant to isoniazid and rifampicin and either to any fluoroquinolone or a second-line injectable agent but not to both. Extensive drug resistance = *Mtb* resistant to isoniazid and rifampicin and to any fluoroquinolone and to at least one of three second-line injectable drugs.

**Table 4:** Type of TB in 30 TB/HIV patients from Minsk according to the DST performed in Belarus and in Denmark, and number of active drugs in treatment regimens.

Type of TB	Minsk^∗^ *n* (%)	Copenhagen^∗∗^ *n* (%)	*p* value	Number of active drugs initially, median (range)		Number of active drugs after 1st change of treatment regimen, median (range)	
				RSPCPT	SRL	RSPCPT	SRL
DS TB	10 (33,3)	12 (40.0)	0.79	4 (4-4)	4 (4-4)	2 (2-3)	2 (2-3)
Isoniazid-resistant TB	2 (6.7)	2 (6.6)	1.00	4 (3-4)	4 (3-4)	5 (4-5)	5 (4-5)
MDR-TB	8 (26.7)	3 (10.0)	0.18	0 (0-5)	0 (0-0)	5 (1-5)	4 (1-5)
preXDR-TB	5 (16.7)	9 (30.0)	0.36	1 (0-1)	1 (0-1)	4 (1-5)	3 (1-4)
XDR-TB	5 (16.7)	4 (13.3)	1.00	0 (0-1)	0 (0-1)	3 (2-3)	3 (1-4)

In Minsk, drug-susceptibility testing (DST) was performed at the Republican Scientific and Practical Center for Pulmonology and TB, Minsk, Belarus. In Copenhagen, DST was performed at the WHO TB Supranational Reference Laboratory, Copenhagen, Denmark. DST = drug-susceptibility test. DS TB = drug-sensitive TB; *Mycobacterium tuberculosis* sensitive to all first-line anti-TB drugs. MDR-TB = multidrug-resistant TB; *Mycobacterium tuberculosis* resistant to both isoniazid and rifampicin. preXDR-TB = preextensive drug-resistant TB; Mycobacterium tuberculosis resistant to isoniazid and rifampicin and either to any fluoroquinolone or a second-line injectable agent but not to both. XDR-TB = extensive drug resistance; *Mycobacterium tuberculosis* resistant to isoniazid and rifampicin and to any fluoroquinolone and to at least one of three second-line injectable drugs. ^∗^As reported. ^∗∗^According to the results of combined phenotypic DST, line probe assay (LPA), and whole-genome sequencing (WGS). In case of discrepancies, the worst result was considered.

## Data Availability

The database contains person-sensitive information and is therefore not publicly available. The TB : HIV Steering Committee encourages the submission of concepts for research proposals. Concepts can be submitted for review using an online research concept (https://www.chip.dk/Studies/TBHIV/Submitresearch-concept). The concept will be evaluated by the Steering Committee for scientific relevance, relevance to the TB : HIV study, design, feasibility, and overlap with already approved projects. All proposers will receive feedback. If approved, a writing group will be established consisting of proposers, members of the Steering Committee, and staff at the coordinating and the statistical centers. The TB : HIV study can be accessed at https://www.chip.dk/Studies/TBHIV, where all relevant documents are available. For submission of a research proposal, please contact Daria Podlekareva (daria.podlekareva@regionh.dk) and Ole Kirk (ole.kirk@regionh.dk). All sequences of *Mycobacterium tuberculosis* obtained within the TB : HIV study have been deposited in the European Nucleotide Archive under project accession number PRJEB38234.
